# Supplementary material for: Systematic review and meta-analysis on juvenile primary spontaneous pneumothorax: Conservative or surgical approach first?
Source: PLoS One. 2021 Apr 30;16(4):e0250929. doi: 10.1371/journal.pone.0250929 (PMC8087103; doi:10.1371/journal.pone.0250929)
Supplement: S1 Table — (DOCX) [file pone.0250929.s001.docx]

**S1 Table. Search strategy.**

| **Final syntax in Embase:** |
| --- |
| (child OR juvenile OR adolescent OR pediatric) AND ((primary AND spontaneous AND pneumothorax OR spontaneous) AND pneumothorax OR pneumothorax) AND (surgical AND approach OR surgery OR operation OR vats) AND ((conservative OR pigtail OR chest) AND tube OR drainage) |
| **Final syntax in Pubmed:** |
| (child OR juvenile OR adolescent OR pediatric) AND (primary spontaneous pneumothorax OR spontaneous pneumothorax OR pneumothorax) AND (surgical approach OR surgery OR operation OR VATS) AND (conservative OR pigtail OR chest tube OR drainage) |
| **Final syntax in Cochrane Database:** |
| Topic: (child OR juvenile OR adolescent OR pediatric) AND (primary spontaneous pneumothorax OR spontaneous pneumothorax OR pneumothorax) AND (surgical approach OR surgery OR operation OR VATS) AND (conservative OR pigtail OR chest tube OR drainage) |
