# Supplementary material for: Systematic review and meta-analysis on juvenile primary spontaneous pneumothorax: Conservative or surgical approach first?
Source: PLoS One. 2021 Apr 30;16(4):e0250929. doi: 10.1371/journal.pone.0250929 (PMC8087103; doi:10.1371/journal.pone.0250929)
Supplement: S2 Table — (DOCX) [file pone.0250929.s002.docx]

| **S2 Table. Quality Assessment.** | | | | | | | | |
| --- | --- | --- | --- | --- | --- | --- | --- | --- |
| Author, year | Bias due to confounding | Selection bias | Bias in the classification of intervention | Deviation from the intended intervention | Bias due to missing data | Bias in the measurement of outcomes | Bias in the selection  of reported results | Overall bias |
| Willams,  2018 [12] | Moderate | Moderate | Serious | Moderate | Low risk | Moderate | Moderate | Serious |
| Soler,  2018 [13] | Moderate | Moderate | Serious | Moderate | Low risk | Moderate | Moderate | Serious |
| Williams,  2017 [6] | Moderate | Moderate | Moderate | Moderate | Low risk | Moderate | Moderate | Moderate |
| Lopez,  2014 [14] | Moderate | Moderate | Serious | Moderate | Moderate | Moderate | Moderate | Serious |
| Seguier-lipszyc, 2011 [3] | Moderate | Moderate | Moderate | Moderate | Low risk | Moderate | Moderate | Moderate |
| Nathan 2010 [15] | Moderate | Moderate | Moderate | Moderate | Low risk | Moderate | Moderate | Moderate |
| Hui,  2006 [16] | Moderate | Moderate | Moderate | Moderate | Low risk | Moderate | Moderate | Moderate |
| Qureshi,  2005 [4] | Moderate | Moderate | Serious | Moderate | Low risk | Moderate | Moderate | Serious |
| Davis,  1993 [17] | Moderate | Moderate | Serious | Moderate | Low risk | Moderate | Moderate | Serious |
