# Supplementary material for: Systematic review and meta-analysis on juvenile primary spontaneous pneumothorax: Conservative or surgical approach first?
Source: PLoS One. 2021 Apr 30;16(4):e0250929. doi: 10.1371/journal.pone.0250929 (PMC8087103; doi:10.1371/journal.pone.0250929)
Supplement: Attachment — Submitted filename: PLOS Review.docx [file pone.0250929.s004.docx]

Summary of the research and overall impression

The authors set out to perform a systematic review of the literature to examine whether early surgical intervention is superior to conservative management for management of primary spontaneous pneumothorax (PSP) in juvenile patients (aged <21 years). The authors performed a meta-analysis of the included studies.

There are currently two major international guidelines on the management of PSP: the British Thoracic Society guidelines, last updated 2010, and the American College of Chest Physicians Delphi consensus statement, published in 2001. Both guidelines pertain to the management of PSP in adults, and these recommendations are frequently extrapolated to juvenile patients. Current guidelines suggest referral for potential surgical intervention in the following instances: 1) second ipsilateral pneumothorax; 2) first contralateral pneumothorax; 3) synchronous bilateral pneumothorax; 4) persistent air leak (>5-7 days); 5) professions at risk (divers, pilots); 6) pregnancy.

There are several options for conservative management, including observation alone, application of supplemental O₂ (100%), needle aspiration, and chest tube drainage.

This study aims to examine the current literature to determine the incidence rate of recurrent primary spontaneous pneumothorax following conservative management and surgical intervention. If surgical intervention greatly reduces the incidence of recurrence, then an argument could be made to offer surgical intervention in first episode PSP.

Strengths

- The study protocol was registered with prospectively with PROSPERO
- A comprehensive database search was performed according to the PRISMA guidelines. Nine studies were included in the final systematic review and meta-analysis.
- No language restrictions were employed in the search
- Two independent reviewers screened title/abstract and extracted the data

Major issues

1. There was significant risk of bias in the included studies, this was documented in the supplementary table (S1) but was not mentioned in the results or considered in the discussion when making a recommendation based on the results.
2. The background incidence of recurrence following conservative management and surgical intervention was not mentioned in the introduction and the calculated incidence of recurrence from the meta-analysis was not reported in the results. These two pieces of information are required for the reader to place the results in context.
3. (Lines 73-76) The search strategy needs to be reviewed and correctly documented; I doubt that the current strategy would yield useful results. I suspect the commas should be the OR operator, and OR should be AND.
4. (Table 1) The authors should document follow up. If the studies all have different lengths of follow up then their reported incidence of recurrence will likely change and therefore meta-analysis may not be appropriate.
5. There is no mention of the risk of major complication following surgical intervention. Any consideration of surgical intervention should weight the risk and benefit of the procedure. If the reduced risk of recurrence is equal to or exceeded by the risk of major morbidity then surgery should not recommended. There should be a brief mention of the risk of major complication in the discussion – this should be well documented in the literature.
6. The authors need to discuss the effect of the various biases present in the systematic review on the strength of their recommendation in the discussion section.

Minor issues

7. (Line 3) The authors have used the acronym ‘PSP’ without defining the term ‘primary spontaneous pneumothorax’
8. The eligibility criteria are not mentioned in the methods section of the abstract (juveniles aged <21)
9. (Lines 39-40) this sentence is grammatically incorrect, I think a word is missing e.g. “Furthermore, no evidence-based pediatric-specific guidelines for spontaneous pneumothorax management exist”
10. (Lines 45-46) these last two sentences could be better written as a single sentence.
11. (Lines 48-52) this statement should clearly define your Population, Intervention, Comparator, Outcome. Intervention and outcome are mentioned, the Population should be better defined (patients aged <21) and the outcomes need to be mentioned (incidence of recurrence and hospital length of stay).
12. (Lines 57-58) previous works don’t need to be cited as evidence of experience with systematic reviews.
13. (Line 104) the authors should mention that the secondary outcome: hospital length of stay was assessed, and state whether mean ± standard deviation or median and interquartile range was used for analysis.
14. (Table 1) It may be worth including gender in the table if reported in the initial studies. It may also be worth breaking down the number of included patients to state how many underwent conservative management and how many underwent surgical intervention.
15. (Line 157) the authors refer to four groups, there should only be two: intervention and comparator.
16. (Line 161-168) it is unclear to me what is meant by “surgical approach after conservative approaches”. Are these patients who failed conservative management due to prolonged air leak, or had recurrent pneumothorax after conservative management?
17. A limitation of the study not mentioned is the small sample size of the included studies. One study, Williams 2017, had a large sample but only reported hospital length of stay. All the studies reporting the primary outcome had <100 patients. The potential effect of this bias on the magnitude of the treatment effect should be discussed.
18. Forest plots need headings

Recommendation

Acceptable for publication with major alterations.

I would be happy to review a revised manuscript.
