# Supplementary material for: Systematic review and meta-analysis on juvenile primary spontaneous pneumothorax: Conservative or surgical approach first?
Source: PLoS One. 2021 Apr 30;16(4):e0250929. doi: 10.1371/journal.pone.0250929 (PMC8087103; doi:10.1371/journal.pone.0250929)
Supplement: Attachment — Submitted filename: Response to Reviewers.docx [file pone.0250929.s005.docx]

**Journal Revised Submissions Cover Letter**

PLOS One

Revised Submission Date: 03/25/2021

Dear Editor,

We would like to thank you for the letter dated 24/02/2021, and the opportunity to resubmit a revised copy of manuscript entitled, " A systematic review and meta-analysis of juvenile primary spontaneous pneumothorax: operation or conservative treatment only? " The reviewers’ thoughtful and kindful comments and suggestions have been carefully revised accordingly. The detailed and revised version has been uploaded with this cover letter.

The manuscript has been revised to response the reviewers’ comments, which are appended alongside our responses to this letter. Furthermore, we need to apologize for minor spelling errors of one author’s name and affiliation of the institute. We have corrected them in this revised copy.

We very much hope the revised manuscript is accepted for publication in *PLOS One*.

Best Regards,

Sincerely yours,

Fu-Huan Huang

Corresponding Author

Fu-Huan Huang, Taipei Medical University Hospital

Address: No. 252, Wuxing St, Xinyi District, Taipei City, 110, Taiwan

E-mail: 191009@h.tmu.edu.tw

Tel: (886)975837299

Major issues

1. There was significant risk of bias in the included studies, this was documented in the supplementary table (S1) but was not mentioned in the results or considered in the discussion when making a recommendation based on the results.

**Response:**

The responses of issue 1 and 6 are integrated here because of their similarities. We have re-evaluated all the studies under ROBINS-I, and it is thought that risk of bias existed. We have described the possibility of bias in the discussion section and mentioned that it could probably weaken the recommendations. Please refer to the discussion section (Line 324-331).

2. The background incidence of recurrence following conservative management and surgical intervention was not mentioned in the introduction and the calculated incidence of recurrence from the meta-analysis was not reported in the results. These two pieces of information are required for the reader to place the results in context.

**Response:**

We added the background incidence of recurrence from the result of the Taiwan nationwide health insurance database (Line 46-47); we also added the calculated result in the text (Line 143-145,180-182).

3. (Lines 73-76) The search strategy needs to be reviewed and correctly documented; I doubt that the current strategy would yield useful results. I suspect the commas should be the OR operator, and OR should be AND.

Response:

We corrected the description of search strategy (Line 73-77 and S1 table).

4. (Table 1) The authors should document follow up. If the studies all have different lengths of follow up then their reported incidence of recurrence will likely change and therefore meta-analysis may not be appropriate.

**Response:**

Although definite follow-up period was only mentioned by half of included studies (four of nine), we added study period of every study into Table 1 in addition to follow-up period. Please see Table 1.

Lower incidence of recurrence could be found from 4 years after first episode of PSP, so at least 4 to 5 years of follow-up or study period might be adequate (Line 319-323).

5. There is no mention of the risk of major complication following surgical intervention. Any consideration of surgical intervention should weight the risk and benefit of the procedure. If the reduced risk of recurrence is equal to or exceeded by the risk of major morbidity then surgery should not recommended. There should be a brief mention of the risk of major complication in the discussion – this should be well documented in the literature.

**Response:**

There was no mention of major complications in surgical intervention group in those studies included for analysis, and we added the descriptions in discussion (Line 238-240). Also, we already mentioned the evidence that VATS was accepted as a much safer and more feasible procedure than open thoracotomy which kind of surgical intervention might cause more complications concerned by physicians (Line 243-246).

6. The authors need to discuss the effect of the various biases present in the systematic review on the strength of their recommendation in the discussion section.

**Response:**

Please refer to the response for issue 1.

Minor issues

7. (Line 3) The authors have used the acronym ‘PSP’ without defining the term ‘primary spontaneous pneumothorax’

**Response:**

We have already corrected it. (Line 3)

8. The eligibility criteria are not mentioned in the methods section of the abstract (juveniles aged <21)

**Response:**

We have already added it in to the criteria. (Line 14)

9. (Lines 39-40) this sentence is grammatically incorrect, I think a word is missing e.g. “Furthermore, no evidence-based pediatric-specific guidelines for spontaneous pneumothorax management exist”

**Response:**

We have already corrected it as suggested. (Line 40)

10. (Lines 45-46) these last two sentences could be better written as a single sentence.

**Response:**

We have already merged these two sentences. (Line 43-46)

11. (Lines 48-52) this statement should clearly define your Population, Intervention, Comparator, Outcome. Intervention and outcome are mentioned, the Population should be better defined (patients aged <21) and the outcomes need to be mentioned (incidence of recurrence and hospital length of stay).

**Response:**

Thanks for this constructive suggestion. We believed there has already been clearly described including parts of PICO after adding the age criteria. (Line 49-53)

12. (Lines 57-58) previous works don’t need to be cited as evidence of experience with systematic reviews.

**Response:**

Thanks for the suggestion. This citation has already been deleted.

13. (Line 104) the authors should mention that the secondary outcome: hospital length of stay was assessed, and state whether mean ± standard deviation or median and interquartile range was used for analysis.

**Response:**

We have already added the description. (Line 104-105,108)

14. (Table 1) It may be worth including gender in the table if reported in the initial studies. It may also be worth breaking down the number of included patients to state how many underwent conservative management and how many underwent surgical intervention.

**Response:**

We have already modified and completed the table as your constructive suggestion. Please see Table 1.

15. (Line 157) the authors refer to four groups, there should only be two: intervention and comparator.

**Response:**

We have already corrected it. (Line 169)

16. (Line 161-168) it is unclear to me what is meant by “surgical approach after conservative approaches”. Are these patients who failed conservative management due to prolonged air leak, or had recurrent pneumothorax after conservative management?

**Response:**

Yes. As your description.

17. A limitation of the study not mentioned is the small sample size of the included studies. One study, Williams 2017, had a large sample but only reported hospital length of stay. All the studies reporting the primary outcome had <100 patients. The potential effect of this bias on the magnitude of the treatment effect should be discussed.

**Response:**

We have added it as one of the limitations for our study. (Line 331-335)

18. Forest plots need headings

Response:

All Forest plots used as figures in this manuscript already have headings in the title of every figure. We re-arranged the location of Figure titles and legends under the guide from formatting example. (Line 129,147,184,195,202) We hoped it might be clear enough.
